# Pathophysiology of sepsis-associated brain dysfunction: an experimental study of cerebral microdialysis and mitochondrial function

**DOI:** 10.1186/cc11961

**Published:** 2013-03-19

**Authors:** P Kurtz, C Vargas-Lopes, C Madeira, I Mello, R Panizzutti, LC Azevedo, FA Bozza

**Affiliations:** 1 Fiocruz, Rio de Janeiro, Brazil; 2 Hospital Sirio e Libanes, São Paulo, Brazil

## Introduction

Pathophysiology of brain dysfunction associated with sepsis is still poorly understood. Potential mechanisms involve oxidative stress, neuroinflammation and blood-brain barrier alterations. Our purpose was to study the metabolic alterations and markers of mitochondrial dysfunction in a clinically relevant model of septic shock.

## Methods

Twelve anesthetized (midazolam/fentanyl/pancuronium), invasively monitored, and mechanically ventilated pigs were allocated to a sham procedure (*n *= 5) or sepsis (*n *= 7), in which peritonitis was induced by intra-abdominal injection of autologous feces. Animals were studied until spontaneous death or for a maximum of 24 hours. In addition to global hemodynamic and laboratory assessment, intracranial pressure and cerebral microdialysis were assessed at baseline, 6, 12, 18 and 24 hours after sepsis induction. After death, brains were removed and brain homogenates were studied to assess markers of mitochondrial dysfunction.

## Results

**Figure 1:**
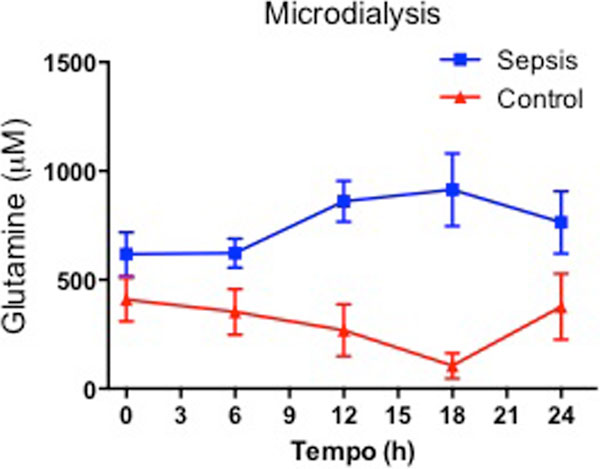


All septic animals developed a hyperdynamic state associated with lower arterial pressure, fever and organ dysfunction in comparison with control animals. In the septic animals, we observed increased brain dialysate glutamin levels at 12, 18 and 24 hours after sepsis induction, as compared with control animals. Moreover, after analyzing homogenates from the frontal cortex, we found higher concentrations of glutamin and glutamate in septic as compared with control animals (85.67 ± 14.98 vs. 28.77 ± 7.0; *P *= 0.01 and 132.1 ± 19.72 vs. 53.33 ± 16.83; *P *= 0.02, respectively). See Figure 1.

## Conclusion

We found higher concentrations of glutamate and glutamin in brain tissues of septic animals as compared with control. Furthermore, glutamin concentrations increased over time in the extracellular space as measured by cerebral microdialysis. These findings suggest an increased excitatory state that is potentially associated with high energy expenditure. However, associations with neuronal injury need further study.

